# Genetic variability studies for tuber yield and yield attributes in Ethiopian released potato (*Solanum tuberosum* L.) varieties

**DOI:** 10.7717/peerj.12860

**Published:** 2022-02-10

**Authors:** Gebrehanna Lemma Tessema, Ali Wassu Mohammed, Desta Tesfaye Abebe

**Affiliations:** 1 Crop Research/Horticulture, Ethiopiopian Institute of Agricultural Research, Holetta Research Centre, Addis Ababa, Ethiopia; 2 Plant Science, Haramaya University, Dire Dawa, Ethiopia, Oromiya, Ethiopia; 3 Crop Research/Horticulture, Ethiopian Institute of Agricultural Research, Addis Ababa, Ethiopia

**Keywords:** *Solanum tuberosum* L., Magnitude of variability, Yield traits, Heritability, Phenotypic coefficient of variation, Genotypic variance, Yield, Phenotypic variance, Genetic advance, Ethiopia

## Abstract

Information on the extent of genetic variability and association among quantitative traits are vital for any crop improvement program and the development of suitable selection strategies. Limited research has been carried out thus far on potato genetic variability and trait association. This study on genetic variability and association among quantitative traits was conducted to assess the extent of genetic variability among yield and agronomic traits to identify superior varieties for the breeding program. To this effect, 20 improved varieties and a local cultivar were planted at two locations in central Ethiopia during the main cropping season of 2017/18 in a randomized complete block design using three replications. Analysis of variance of tuber yield and yield traits at each location and over locations, revealed the existence of highly significant (*P* < 0.01) differences among varieties in all agronomic and yield traits. Phenotypic coefficient of variation values ranged from 0.75% (specific gravity) to 32.22% (total starch yield) while the genotypic coefficient of variation values ranged between 0.70% (specific gravity) to 30.22% (total starch yield). Maximum difference between phenotypic and genotypic coefficient of variation values were noted for stem number, average tuber number, average tuber weight, number of leaves per plant and tuber yield. Hence, these traits are substantially influenced by the physiological status of the seed tuber at planting and by the environment, post emergence. Range of variability for most of the traits was high, indicating ample scope for selection and improvement in these traits. The estimated values for broad sense heritability and genetic advance, as percent of mean, ranged from 33.52% to 98.66% and 1.35% to 58.26%, respectively. All the traits had high heritability values, except average tuber number per hill, days to physiological maturity, average tuber weight and number of leaves per plant with moderate heritability values.

## Introduction

Potato (*Solanum tuberosum*) is an important staple food and cash crop domesticated around 8,000 years ago in the Andes Mountains of South America (International Potato Centre, 2019). Worldwide it is the third most important food crop following rice and wheat in terms of human consumption. It is grown by around 161 countries on an area of 25 million hectares. The world average yield is about 19.44 ton/hectare with total production exceeding 487 million tons in 2017 (FAOSTAT, 2019). Potato has the great potential for sustainable food supply and is an important option for food security in many developing nations. In Africa, however, the average yield lies between six to 12 tons per hectare which is absolutely low compared to the developed world average of 35 to 45 tons per hectare (FAO, 2009; Devaux, Kromann & Ortiz, 2014; Mihovilovich et al., 2017). Shortages of improved varieties (Gebremedhin, 2013) preferred by most farmers, the use of low-quality seed tubers infected with viruses or other pathogens (International Potato Centre, 2018; Sharma et al., 2018; Mihovilovich et al., 2017), and repeated use of local cultivars for an unlimited period without seed or variety renewal (Kolech et al., 2015) had contributed for the low average tuber yield.

Changing this situation requires the development of high-yielding varieties adaptable to wide agro-ecologies and preferred by both growers and consumers. This, in turn, requires knowledge on the genetic components of traits and the extent of genetic variability among improved varieties and widely grown local varieties. Such information will allow plant breeders to predict the response to the selection of breeding programs (Waqar-ul-haq et al., 2008; Bulent, Engin & Seymus, 2013). Allard (1960) designated variability as differences among the individuals due to the alterations in their genetic makeup or the environment in which they are grown. Genetic variability due to the inherent genetic makeup among individuals within a population has an immense importance for plant breeding program. It allows proper diversity management to produce consistent genetic gain in the performance of the plant and buffer periodic instabilities (Sharma, 1998).

Genetic variability of potato clones could be due to either additive, dominant or epistatic types of genetic action owing to their stable genetic structure and transfer to the next generation. This is because in non-inbred species like potato, the heritability is always the summation of additive and dominance genetic variance (van Eck, 2007). Ozturk & Yildirim (2014) reported a moderate to high level heritability values for quantitative characters like plant height, leaf width, leaf length, single tuber weight, tuber yield and starch content from a study carried on 21 potato genotypes. Tripura et al. (2016) reported high broad-sense heritability together with genetic advance for the following plant traits: tuber yield, plant height, tuber number per plant, tuber weight per plant, tuber breadth, and leaf number per plant, whereas Fekadu, Petros & Zelleke (2013) reported low heritability and genetic advance values in potato for the days to emergence, flowering and maturity, stem number, tuber yield, number of tubers, harvest index, medium tuber percentage, and biomass yield characters.

Estimating broad-sense heritability (H^2^), genetic coefficient of variation as well as genetic advance would be helpful to plant breeders to implement selection in breeding programs (Johnson, Robinson & Comstock, 1955). Estimates of heritability based on growing potato genotypes at multiple locations for several years will support potato breeders to decide the breeding strategy that should be followed. Mishra et al. (2006) noticed that high heritability associated with high genetic advance would be used as a clue in most selection programs. Performing principal component analysis on trait of the varieties or populations displaying variability helps to reduce any redundancy in the component variables (Placide et al., 2015).

The national potato research program in Ethiopia needs to improve the crop’s yield potential, important agronomic traits as well disease resistance, and quality traits that are key to meeting consumer preferences. Moreover, knowledge on the degree of genetic variability present among genotypes and the association of quantitative characters with yield is vital for any crop improvement program and also to develop suitable selection strategies (Ene et al., 2016). On the other hand, the estimation of genetic variances helps plant breeders to choose the most efficient breeding design for improving crops with the existing resources (Oloyede-Kamiyo, Ajala & Akoroda, 2014). Such information is scanty owing to the limited work done by the Ethiopian potato breeding program within the existing genetic pool in the country. Therefore, it is necessary to study genetic variability in the yield potential, disease tolerance, and other important traits. The present study was carried to investigate and estimate the nature and extent of variability in yield and agronomic traits among 20 released varieties and one local cultivar and thereby identify superior varieties for a breeding program.

## Materials and Methods

### Experimental materials and sites

The present study was carried out at two locations in the central highlands of Ethiopia, Holetta Research Centre and Adaberga sub-station research fields for a single cropping season (Table 1). The experiment consisted of 20 released varieties and one local potato cultivar (Table S1). The experiment was laid out in a randomized complete block design replicated three times. Analysis of variance for each location and across locations was done using SAS software version 9.3 (SAS Institute, Cary, NC, USA) as per the procedure indicated for the design using a general linear model (GLM) (Gomez & Gomez, 1984).

**Table 1:** Geographic coordinates and weather condition data of Holetta research centre and Adaberga research sub-station.

Parameters	Description	Locations (sites)	
		Holetta	Adaberga
Coordinates	Latitude	09°00′N	09°16′N
	Longitude	38°29′E	38°23′E
	Altitude (m)	2,400	2,500
Weather conditions*	Rainfall (mm)	5.74	9.23
	Minimum temperature (°C)	9.26	8.85
	Maximum temperature (°C)	22.18	21.10

**Notes:**

* Weather condition data was present for five months (June–October, 2017) of a single cropping season. Maximum temperature (°C) 22.18 21.10

*Source*: (Holetta Agricultural Research Centre, 2010 & 2017, unpublished data).

### Data collection and measurement for the traits

Data collection and measurement of yield and agronomic traits (days to 50% flowering, days to physiological maturity, number of leaves per plant, plant height, stem number per hill, average tuber number per hill, average tuber weight, total tuber yield, marketable tuber yield, specific gravity, dry matter content, starch content percentage and total starch yield) were under taken based on the procedures indicated by Lemma, Wassu & Tesfaye (2020).

### Statistical methods

For each quantitative attribute, phenotypic and genotypic variability were estimated using variances and coefficient of variations using the procedure suggested by Burton & de Vane (1953).

${\rm Genotypic\, variance\, (\sigma^2g)}= \displaystyle{{Mg - Me} \over r}$

where σ^2^_g_ = genotypic variance

M_g_ = mean square of genotype

M_e_ = mean square of error; r = number of replications; Phenotypic Variance (σ^2^_p_) = σ^2^_g_ + σ^2^_e_

where, σ^2^_g_ = Genotypic variance; σ^2^_e_ = Environmental variance; σ^2^_p_ = phenotypic variance

$\rm PCV={\left(\sqrt {\sigma_p^2}\over {\overline{x}}\right)}\times {100}$

$\rm GCV={\left(\sqrt {\sigma_g^2}\over{\overline{x}}\right)}\times {100}$

where PCV = Phenotypic coefficient of variation

GCV = Genotypic coefficient of variation

${\bar {\rm x}}$ **=** population mean of the character being evaluated.

PCV and GCV values were classified as low, moderate, and high according to Sivasubramanian & Menon (1973) as 0 up to 10% = Low, 10 up to 20 = Moderate and above 20 = High.

#### Heritability and genetic advance

Broad sense heritability values were estimated for each location using the formula adopted by Falconer & Mackay (1996) as follows

$H^2= \displaystyle{{{\rm \sigma }2{\rm g}} \over {{\rm \sigma }2{\rm p}}}\; \times {100}$

where H^2^ is broad-sense heritability, σ^2^p-phenotypic variance

σ^2^g-genotypic variance

The heritability percentage was categorized as low, moderate, and high, as suggested by Robinson, Comstock & Harvey (1949):-

$\rm 0 \,to \,30{\%}= Low,\, 30{\%}\, to\, 60{\%}= Moderate,\, {>}60{\%}= High$

#### Expected genetic advance under selection

Genetic advance in absolute units (GA) and percent of the mean (GAM), assuming selection of superior 5% of the genotypes, were estimated for each location based on the methods illustrated by Johnson, Robinson & Comstock (1955):

$\rm {GA} = {{K} * {SD_p} * {H^2}}$

where, GA = Genetic advance

SDp = Phenotypic standard deviation on mean basis;

H^2^ = Heritability in the broad sense.

K = the standardized selection differential at 5% selection intensity (K = 2.063).

#### Genetic advance as percent of mean

Genetic advance as percent of mean was estimated as follows:

$\rm {GAM}=\displaystyle{{\rm GA} \over \overline x}\times{100}$

where GAM = Genetic advance as percent of mean

The GAM values were grouped in to low, moderate, and high categories, as suggested by Johnson, Robinson & Comstock (1955):

$\rm 10{\%}=Low,\, 10{\%}\, to\, 20{\%}= Moderate,\, {>}20{\%}=High$

### Estimates of variability components over locations

The computation of genotypic and phenotypic variance across locations considered the expected mean squares from combined analysis of variance and the σ^2^P, σ^2^G and H^2^ = phenotypic variance, genotypic variance and heritability in broad sense were determined as indicated in Table 2 as proposed by Jalal & Ahmad (2012). Principal component analysis (PCA) was conducted to reduce the observed agronomic and yield traits as suggested by Amy & Pritts (1991). The study considered the main component of maximum eigenvalue of up to 1.0 to estimate the variability in potato varieties studied.

**Table 2:** Estimation of variance components and broad sense heritability on combined analysis of variance over locations basis.

Genotypic parameter	Symbol	Determination method
Error variance	σ^2^E	MSE
Location variance	σ^2^L	(MSL–MSGL)/rg
Genotypic variance	σ^2^G	(MSG–MSGL)/rl
G × L interaction variance	σ^2^GL	(MSGL-MSE)/r
Phenotypic variance	σ^2^P	σ^2^G+ (σ^2^GE/l) + (σ^2^E/rl)
Broad sense heritability	H^2^	σ^2^G/σ^2^P

**Note:**

σ^2^E, error variance; σ^2^L, location variance; σ^2^G, genotypic variance; σ^2^GL, genotype × location interaction variance; σ^2^P, phenotypic variance; H^2^, heritability in broad sense; MSE, mean square error; MSL, mean square location; MSG, mean square genotype; MSGL, mean square genotype × location interaction; and rl, replication by location.

## Results

Results of the variance analysis indicated adequate variability among varieties over all the traits considered (Table 3). The analyzed varieties were widely variable over the analyzed characteristics. Tuber yield varied significantly among improved varieties and local cultivars (13.8 to 32.8 t ha^−1^). The maximum mean values of tuber yield, specific gravity, dry matter content, starch content and total starch yield were recorded from the variety Belete while the lowest mean value of all these traits was recorded from the variety Menagesha (Table 4).

**Table 3:** Analysis of variance for agronomic and yield traits of 21 potato varieties tested at two locations in central Ethiopia in 2017.

Trait	Rep (L) (4)	Variety (V) (20)	Location (L) (1)	L × V (20)	Error (80)	CV (%)
Days to 50% flowering	10.01	61.24**	5.37	0.82	9.68	5.22
Days to physiological maturity	49.25	160.96**	1176.36**	103.82**	10.72	3.30
Number of leaves per plant	8.76	219.16**	3618.22**	117.17**	16.31	9.89
Plant height (cm),	138.97	635.76**	18112.8**	120.05**	16.46	6.84
Stem number per hill	1.15	8.53**	65.29**	2.90**	0.24	11.12
Average tuber number per hill	4.96	25.15**	48.52**	16.72**	2.26	13.35
Average tuber weight (g)	21.24	600.91**	4538.05**	378.75**	21.27	8.95
Total tuber yield (t ha^−1^)	16.99	218.57**	2556.72**	49.37**	4.39	8.29
Marketable tuber yield (t ha^−1^)	15.02	205.99**	1927.87**	44.39**	3.82	9.13
Unmarketable tuber yield (t ha^−1^)	2.63	6.44**	44.32**	1.77**	0.38	15.99
Specific gravity (gcm^−3^)	0.0002	0.0004**	0.008**	0.00005	0.00004	0.61
Dry matter content (%)	2.14	20.54**	321.44**	2.23	1.50	5.60
Starch content percent (g 100 g^−1^)	5.62	30.67**	284.76**	4.64*	2.35	10.74
Total starch yield (t ha^−1^)	1.15	8.13**	9.81**	1.03**	0.20	12.36

**Note:**

*, **, significant at *P* = 0.05 and *P* < 0.01, respectively. Rep = replication, CV (%) = coefficient of variation in percent, numbers in the parenthesis are degrees of freedom.

**Table 4:** Mean performances of 21 potato varieties for tuber yield and other traits evaluated at Holetta and Adaberga in 2017.

	Traits												
Variety	DF	DM	NLP	PH (cm)	SN	ATN	ATW	TTY	MTY	SG	DMC	SC	TSY
Dagim	59.7^b-f^	102.0^cd^	41.4^cde^	57.0^fg^	4.1^ef^	8.1^hi^	70.0^a^	23.4^i^	19.9^e^	1.087^bcd^	22.042^b-g^	14.537^b-f^	3.378^fg^
Bubu	65.2^a^	105.2^abc^	40.5^ef^	66.8^bc^	5.9^c^	11.9^cde^	54.6^cde^	28.9^c-f^	24.7^cde^	1.092^b^	23.375^bc^	15.982^bc^	4.520^bcd^
Belete	57.7^d-g^	104.3^a-d^	43.1^cde^	67.3^bc^	4.4^de^	10.9^ef^	65.0^ab^	32.8^a^	29.1^a^	1.102^a^	25.417^a^	18.430^a^	5.887^a^
Gudene	60.7^b-e^	96.0^fg^	50.5^ab^	71.6^ab^	6.5^b^	14.6^ab^	48.6^efg^	31.8^ab^	26.3^bc^	1.090^bcd^	22.833^bcd^	15.397^bc^	4.815^b^
Challa	56.3^fg^	107.2^ab^	41.5^cde^	61.5_def_	3.0^fg^	10.6^efg^	56.1^cd^	26.3^fgh^	23.4^de^	1.092^b^	23.625^b^	16.408^b^	4.203^cde^
Marachere	56.7^efg^	108.0^a^	34.4^gh^	42.0^ij^	2.8^hi^	10.6^efg^	35.6^j^	20.4^j^	17.3^gh^	1.080^d^	20.292^i^	12.172^g^	2.423^h^
Shenkolla	61.0^bcd^	103.2^bcd^	51.9^a^	69.2^ab^	7.3^a^	13.2^bcd^	50.1^def^	29.6^b-e^	24.1^cde^	1.087^bcd^	22.625^b-e^	15.117^bc^	4.330^b-e^
Gabissa	59.8^b-f^	93.7f^gh^	35.9^fg^	60.5^ef^	5.4^c^	13.4^bc^	53.7^cde^	31.6^abc^	27.3^ab^	1.088^bcd^	22.500^b-f^	15.018^bcd^	4.663^bc^
Gera	58.0^c-g^	101.3^cd^	45.7^b-e^	71.5^ab^	4.3^de^	10.7^efg^	64.1^b^	29.6^b-e^	27.2^ab^	1.082^cd^	20.917^f-i^	12.993^d-g^	3.812^efg^
Jalene	57.7^c-g^	97.0^ef^	45.4^b-e^	52.9^gh^	4.5^de^	11.5^c-f^	44.6^fgh^	23.8^hi^	19.1^fg^	1.090^bc^	22.792^bcd^	15.560^bc^	3.558^fg^
Gorebella	54.7^g^	100.3^de^	46.6^ab^	74.2^a^	4.8^d^	9.5^fgh^	66.9^ab^	30.4^a-d^	27.4^ab^	1.089^bc^	22.583^b-e^	15.217^bc^	4.585^bc^
Guassa	62.2^abc^	101.0^cde^	34.6^gh^	44.3^i^	3.2^gh^	7.3^i^	58.5^c^	17.8^k^	15.1^hi^	1.090^bc^	22.833^bcd^	15.475^bc^	2.635^h^
Zengena	60.8^b-f^	103.5^bcd^	44.4^cde^	63.2^cde^	3.3^gh^	9.5^fgh^	36.5^ij^	15.9^kl^	12.4^j^	1.088^bcd^	21.875^c-i^	14.275^c-f^	2.260^hi^
Zemen	62.5^ab^	100.3^de^	42.0^cde^	66.6^bcd^	5.7^c^	11.4^c-f^	44.1^gh^	25.8^ghi^	22.1^e^	1.087^bcd^	22.417^b-f^	14.822^b-e^	3.797^efg^
Bedassa	61.8^a-d^	92.0^gh^	41.2^cde^	57.4^fg^	4.1^def^	15.7^a^	44.5^fgh^	30.7^a-d^	25.6^bcd^	1.082^cd^	20.625^ghi^	12.770^efg^	3.812^efg^
Chiro	60.7^b-e^	95.0^fgh^	31.5^gh^	63.9^cde^	4.1^def^	13.1^bcd^	52.0^de^	28.1^d-g^	24.9^bcd^	1.083^bcd^	21.625^d-i^	14.108^c-g^	3.845^efg^
Wechecha	61.7^a-d^	93.3^fgh^	35.8^fg^	45.0^i^	4.5^de^	11.3^def^	35.7^j^	17.2^k^	13.3^ij^	1.082^cd^	21.042^e-i^	12.857^efg^	2.205^hi^
Menagesha	51.0^h^	100.3^de^	30.5^h^	38.9^j^	2.5^i^	8.8^ghi^	53.7^cde^	16.1^kl^	13.9^ij^	1.057^e^	15.708^j^	6.700^h^	1.068^j^
Awash	61.3^a-d^	91.0^h^	32.8^gh^	50.7^h^	3.4^gh^	12.2^cde^	54.2^cde^	28.9^c-f^	23.8^cde^	1.088^bcd^	21.958^b-h^	14.397^b-f^	4.098^c-f^
AL-624	60.7^b-e^	93.3^fgh^	46.1^bcd^	63.2^cde^	4.5^de^	11.6^cde^	52.6^cde^	27.7^efg^	23.9^cde^	1.088^bcd^	22.250^b-g^	14.587^b-f^	3.997^def^
Nech Abeba	63.2^ab^	93.8^fgh^	40.7^def^	58.9^ef^	4.5^de^	10.7^efg^	41.5^hi^	13.8^l^	8.4^k^	1.080^d^	20.375^hi^	12.607^fg^	1.747^i^
Grand Mean	59.63	99.14	40.79	59.36	4.43	11.26	51.55	25.26	21.39	1.09	21.89	14.26	3.60

**Note:**

DF, days to 50% flowering; DM, days to physiological maturity; NLP, number of leaves per plant, PH, plant height (cm); SN, stem number per hill; ATN, average tuber number per hill; ATW, average tuber weight(g/tuber); TTY, total tuber yield (t ha^−1^); MTY, marketable tuber yield (t ha^−1^); SG, specific gravity (gcm^−3^); DMC, dry matter content (%); SC, starch content percent (g 100 g^−1^); TSY, total starch yield (t ha^−1^).

The results of estimates of variability components (phenotypic and genotypic coefficient of variations, heritability, and genetic advance) for each location are presented in Table 5. The estimates of the variability of components across sites are shown in Table 6.

**Table 5:** Estimates of variability components for agronomic traits of 21 potato varieties at Holetta and Adaberga in 2017.

	Holetta							Adaberga						
Trait	σ^2^g	σ^2^p	GCV (%)	PCV (%)	H^2^ (%)	GA (5%)	GAM (%)	σ^2^g	σ^2^p	GCV (%)	PCV (%)	H^2^ (%)	GA	GAM (%)
DF	8.06	11.01	4.74	5.54	74.92	5.01	8.36	6.17	9.68	4.18	5.24	63.74	4.09	6.87
DM	71.98	76.57	8.83	9.11	94.17	16.95	17.64	9.13	11.70	2.96	3.35	78.08	5.50	5.38
NLP	81.40	86.61	19.55	20.17	93.98	18.02	39.04	19.83	25.50	12.57	14.25	77.79	8.09	22.84
PH	158.86	163.19	17.66	17.90	97.34	25.62	35.90	82.11	88.74	19.13	19.89	92.52	17.95	37.90
SN	3.15	3.23	34.45	34.88	97.52	3.61	70.07	0.50	0.58	19.11	20.60	86.04	1.35	36.51
ATN	5.49	6.26	19.72	21.06	87.75	4.52	38.06	6.96	7.70	24.79	26.07	90.39	5.17	48.55
ATW	150.94	156.59	21.35	21.74	96.40	24.85	43.18	161.43	169.96	27.90	28.62	94.98	25.51	56.00
TTY	64.45	66.43	26.97	27.38	96.60	16.29	54.72	21.94	22.89	22.56	23.05	95.86	9.45	45.51
MTY	58.38	60.20	30.20	30.66	96.98	15.50	61.26	22.54	23.26	27.16	27.59	96.88	9.63	55.07
SG	0.00	0.00	0.62	0.71	84.74	0.01	1.11	0.00	0.00	0.83	0.90	84.59	0.02	1.57
DMC	2.51	2.98	7.81	8.51	84.25	3.00	14.77	4.08	4.61	8.60	9.14	88.47	3.91	16.66
SC	2.76	3.27	13.02	14.18	84.30	3.14	24.63	7.45	8.50	17.31	18.49	87.64	5.26	33.39
TSY	1.79	1.87	34.51	35.23	96.26	2.70	69.62	1.13	1.18	31.97	32.75	95.31	2.14	64.30

**Note:**

σ^2^g, σ^2^p, Genotypic variance and phenotypic variance, respectively; GCV (%) and PCV (%), Genotypic and phenotypic coefficient of variation, (%); H^2^%, Heritability; GA, Genetic advance; and GAM (%), Genetic advance as percentage of mean. DF, days to 50% flowering; DM, days to physiological maturity; NLP, number of leaves per plant; PH, plant height (cm); SN, stem number per hill; ATN, average tuber number per hill; ATW, average tuber weight (g/tuber); TTY, total tuber yield (t ha^−1^); MTY, marketable tuber yield (t ha^−1^); SG, specific gravity (gcm^-3^); DMC, dry matter content (%); SC, starch content percent (g 100 g^−1^); TSY, total starch yield (t ha^−1^).

**Table 6:** Estimates of variability components over two locations (Holetta and Adaberga) for different traits of 21 potato varieties in 2017.

Trait	Range	Mean	σ^2^gl	σ^2^l	σ^2^g	GCV (%)	PCV (%)	H^2^ (%)	GA	GAM
Days to 50% flowering	51.0–65.2	59.63	−2.95	0.07	10.06	5.32	5.36	98.66	6.50	10.90
Days to physiological maturity	91.0–108.0	99.14	31.03	17.02	9.65	3.11	5.22	35.50	3.79	3.83
Number of leaves per plant	30.5–51.9	40.79	33.62	55.57	17.00	10.11	14.82	46.54	5.80	14.22
Plant height (cm)	38.9–74.2	59.36	34.53	285.60	85.95	15.62	17.34	81.12	17.23	29.02
Stem number per hill	2.5–7.3	4.43	0.89	0.99	0.94	21.87	26.92	66.00	1.62	36.65
Average tuber number per hill	7.3–15.7	11.26	4.82	0.50	1.40	10.53	18.18	33.52	1.42	12.57
Average tuber weight (g)	35.6–70.0	51.55	119.16	66.02	37.02	11.80	19.41	36.97	7.63	14.81
Total tuber yield (t ha^−1^)	13.8–32.9	25.26	14.99	39.80	28.19	21.02	23.89	77.41	9.64	38.16
Marketable tuber yield (t ha^−1^)	8.4–29.1	21.39	13.52	29.90	26.94	24.26	27.39	78.45	9.48	44.33
Specific gravity (gcm^−3^)	1.057–1.102	1.085	0.00	0.00	0.00	0.70	0.75	87.50	0.01	1.35
Dry matter content (%)	15.7–25.4	21.89	0.24	5.07	3.05	7.98	8.45	89.14	3.40	15.54
Starch content percent (g 100 g^−1^)	6.7–18.4	14.26	0.76	4.45	2.54	14.61	15.85	84.87	3.96	27.76
Total starch yield (t ha^−1^)	1.1–5.9	3.60	0.28	0.14	1.06	30.22	32.33	87.33	2.10	58.26

**Note:**

σ^2^gl, variance for genotype x location; σ^2^l, variance for location; σ^2^g, genotypic variance; GCV (%), genotypic coefficient of variation in percent; PCV (%), phenotypic coefficient of variation in percent; H^2^ (%), heritability in broad sense in percent; GA, genetic advance at 5% selection intensity; and GAM, genetic advance as percent of mean at 5% selection intensity.

### Phenotypic and genotypic variation

At Holetta, the estimated phenotypic (PCV) and genotypic (GCV) coefficient of variations of specific gravity and total starch yield of the different potato genotypes studied ranged between (0.71% to 35.23% and 0.62% to 34.51%), respectively. At Adaberga the PCV and GCV values of specific gravity and total starch yield ranged between (0.91% to 32.75% and 0.83% to 31.97%), respectively (Table 5). The over locations PCV values range from 0.75% (specific gravity) to 32.22% (total starch yield) while the GCV values ranged from 0.70% (specific gravity) to 30.22% (total starch yield) (Table 6). The results of the phenotypic variance were in general higher than the genotypic variance for all characters studied.

The difference between PCV and GCV was the most pronounced for the traits stem number, average tuber number, average tuber weight, number of leaves per plant, tuber yield. Therefore, we speculated that these traits are substantially influenced by the growing environments.

### Estimates of heritability and genetic advance

The estimated values of H^2^ and GAM ranged from 33.52% (average tuber number per hill) to 98.66% (days to 50% flowering) and 1.35% (specific gravity) to 58.26% (total starch yield), respectively (Table 6). All the traits had high heritability values but average tuber number per hill, days to physiological maturity, average tuber weight, and the number of leaves per plant. Highest GAM was recorded for total starch yield, starch content percent, total and marketable tuber yields, plant height, and the number of stems per hill. High heritability coupled with high GAM was recorded from all the traits with high GAM, meaning that a simple breeding method could be employed to advance these traits. Comparatively moderate heritability with low and moderate GAM was recorded for days to physiological maturity, the number of leaves per plant, average tuber number per hill, and average tuber weight per plant, suggesting non-additive gene action in their control: hence complex breeding methods might be recommended improving potato yield through these traits.

### Principal component analysis

The principal component (PC) analysis was used to combine the observed traits into four main components that have eigenvalues above 1.0 and was able to explain the tested variability at 87.53% (Table 7). PC1 with the eigenvalue 5.7 explained 47.53% of the total variance, PC2 with the eigenvalue 2.1 contributed to 17.73% of the total variance, PC3 with the eigenvalue of 1.6 contributed to 13.57% of the total variance and PC4 with the eigenvalue 1.0 contributed to 8.70% among the 20 potato varieties and one local cultivar. The first principal component is strongly positively correlated with plant height, total tuber yield and total starch yield. The cumulative variance of 87.53% by the first four axes indicated that total tuber yield, total starch yield ton per hectare, and plant height contributed to the larger share of the observed variations among potato varieties and could effectively be used for selection. Other phenotypes, such as the number of “days to 50% flowering”, “average tuber number per hill”, and “marketable tuber yield ton per hectare” were also important, as they negatively affect the yield (Table 7).

**Table 7:** Eigen vectors, eigen values, variation explained (%) and cumulative variance (%) of the first four principal components related to 11 traits in 20 improved potato varieties and one local cultivar.

Traits	PC1	PC2	PC3	PC4
Days to 50% flowering	0.1331	−0.4079	0.3953	−0.3528
Days to physiological maturity	0.0063	0.4828	0.2759	0.2517
Number of leaves per plant	0.2665	−0.0006	0.2481	0.5974
Plant height (cm)	0.3509	0.0199	0.0688	0.3316
Stem number per plant	0.2970	−0.2747	0.1572	0.2993
Average tuber number per hill	0.2090	−0.5039	−0.2466	0.0433
Average tuber weight (g/tuber)	0.1722	0.4613	−0.2620	−0.1214
Total tuber yield (t ha^−1^)	0.3722	0.0014	−0.3390	−0.0589
Marketable tuber yield (t ha^−1^)	0.3562	0.0866	−0.3715	−0.0655
Dry matter content (%)	0.3306	0.1229	0.3371	−0.3368
Total starch yield (t ha^−1^)	0.3990	0.0658	−0.1669	−0.1377
Eigenvalue	5.7040	2.1273	1.6280	1.0441
Variation explained (%)	0.4753	0.1773	0.1357	0.0870
Cumulative variance (%)	0.4753	0.6526	0.7883	0.8753

**Note:**

PC, Principal component.

## Discussion

The observed genetic variability among the evaluated varieties in the present study indicated the presence of breeding lines that can be used as parental materials for the potato improvement program (Table 4). Four varieties (Gudene, Gabissa, Gorebella, and Bedassa) had maximum total tuber yield t ha^−1^ without significant differences from the highest yielding variety (Belete) but significantly different from the other varieties (Table 4). Two varieties (Zengena and Menagesha) had a low total tuber yield t ha^−1^ which is on par with the lowest yielding (local cultivar). Yeshanew, Tsegaw & Seyoum (2010) supports this argument: they have reported tuber yield of 15.54 to 34.37 t ha^−1^ among potato varieties in northwestern Ethiopia. Gebreselassie, Mohammed & Shimelis (2016) also reported a total tuber yield variation of 18.34 to 48.29, 26.71 to 43.50 and 17.70 to 56.52 t ha^−1^ in Haramaya, Arbarakete and Hirna, respectively in a study conducted in eastern Ethiopia. Based on yield superiority and important traits identified, the breeding program could use those prospective varieties for future breeding programs as a parent for future potato improvement works.

### Phenotypic and genotypic variation

The range of PCV and GCV variability for most of the characters were high, indicating the presence of sufficient scope for selection and improvement in these characters, including tuber yield t ha^−1^. Similarly, maximum PCV and GCV values were reported by Abraham (2013) and Fekadu, Petros & Zelleke (2013) and Singh (2008) for tuber yield and other yield-related traits. These results agreed with Tripura et al. (2016), who reported high PCV and GCV values for tuber yield and additional yield-related traits as average tuber weight, tuber number, tuber breadth, and tuber yield in potato. Low PCV and GCV values were recorded for some tuber quality traits *viz*., specific gravity and dry matter content which is due to the fact that most tuber quality traits like tuber specific gravity and dry matter content are less influenced by environments (Singh et al., 2006). Likewise, Chepkoech et al. (2018) reported moderate PCV values and low GCV values in plant height, stem number, tuber number, and tuber weight traits. Low to moderate PCV and GCV values were also reported for tuber length (mm), single tuber weight (g), plant height (cm), branch number, lateral leaflet, and the number of tubers per plant.

### Estimates of heritability and genetic advance

In the present study, heritability estimates were high for most of the studied traits as categorized (Low <30%; Moderate 30–60%; High >60%) by Robinson, Comstock & Harvey (1949) and Johnson, Robinson & Comstock (1955). The presence of high heritability in most traits indicates a lower environmental influence. Similar results were reported by Panja et al. (2016). Johnson, Robinson & Comstock (1955) categorized GAM values as Low (0–10), Moderate (10–20), and High (≥20). In the present study, results of analysis of GAM of all the studied traits had high to moderate values except specific gravity and days to physiological maturity that exhibited low GAM (1.35 and 3.83), respectively.

High value of H^2^ and GAM indicated the existence of genetic variation in potato varieties which could be an opportunity for further improvement of potato. Similarly, Tripura et al. (2016) reported high heritability values coupled with high genetic advance for an average tuber weight plant^−1^, single tuber weight, and tuber breadth. Similar reports for total tuber yield (kg per plot) were reported by Rangare & Rangare (2017) and Panigrahi et al. (2017). Likewise, Ozturk & Yildirim (2014) published moderate to high heritability for plant height, leaf width, leaf length, single tuber weight, plant yield, and starch content in potato. Low H^2^ and GA values were reported by Fekadu, Petros & Zelleke (2013) for days to emergence, days to flowering, days to maturity, stem number, tuber yield, number of tubers, harvest index, medium tuber percentage, and biomass yield in potato.

### Principal component analysis

The results of principal component analysis (PCA) for the studied traits are presented in Table 7. In the present study, the first three traits (plant height, total tuber yield and total starch yield) explained 47.53% of the variation. The result indicated that the cumulative variance of 87.53% was explained by the first four axes (Table 7). Afuape, Okocha & Nijoku (2011) reported a cumulative variance of 76.00% for the first three axes in evaluating twenty-one sweet potato genotypes. Koussao et al. (2014) identified four principal components, which accounted for 67.22% of the total variation among the accessions. Placide et al. (2015) also used PCA to study the fifty-four sweet potato genotypes and found that the first seven PCs explain the cumulative variance of 77.83%. Rahanjeng & Rahayuningsih (2017) reported 79% of variability using sixty-two sweet potato accessions. In our study the first four principal components explained 87.53% of the variability. This value is within the range mentioned in earlier studies.

## Conclusions

In general, this study found diverse genetic variability estimates among potato varieties studied. The agronomic characters of potato varieties showed high genotypic coefficient of variation and phenotypic coefficient of variation values, high estimation of broad-sense heritability values for most traits some coupled with genetic advance as percent of mean. Hence, this information and the identified varieties and traits could be used for future potato breeding programs in the country.

## Supplemental Information

10.7717/peerj.12860/supp-1 Supplemental Information 1 Climate data for single location as raw data.

10.7717/peerj.12860/supp-2 Supplemental Information 2 Raw data.

10.7717/peerj.12860/supp-3 Supplemental Information 3 List of experimental materials included in the study at Holetta and Adaberga in 2017.

## Additional Information and Declarations

### Data Availability

The following information was supplied regarding data availability: The raw data are available in the Supplemental Files.
