# Supplementary material for: Genetic variability studies for tuber yield and yield attributes in Ethiopian released potato (Solanum tuberosum L.) varieties
Source: PeerJ. 2022 Feb 10;10:e12860. doi: 10.7717/peerj.12860 (PMC8841037; doi:10.7717/peerj.12860)
Supplement: Supplemental Information 3 [file peerj-10-12860-s003.docx]

Supplementary Table 1. List of experimental materials included in the study at Holetta and Adaberga in 2017

| No. | Variety | Accession  Code | Year of release | Breeding centre* | Recommended  altitude (m.a.s.l) |
| --- | --- | --- | --- | --- | --- |
| 1 | Dagim | CIP-396004.337 | 2013 | ADARC | 2000-2800 |
| 2 | Bubu | CIP-384321.3 | 2011 | HU | 1700-2000 |
| 3 | Belete | CIP-393371.58 | 2009 | HARC | 1600-2800 |
| 4 | Gudene | CIP-386423.13 | 2006 | HARC | 1600-2800 |
| 5 | Challa | CIP 387412-2 | 2005 | HU | 1700-2000 |
| 6 | Mara chare | CIP 389701-3 | 2005 | AwARC | 1700-2700 |
| 7 | Shenkolla | KP- 90134.5 | 2005 | AwARC | 1700-2700 |
| 8 | Gabissa | CIP 3870-96-11 | 2005 | HU | 1700-2000 |
| 9 | Gera | KP-90134.2 | 2003 | ShARC | 2700-3200 |
| 10 | Jalene | CIP-384321.19 | 2002 | HARC | 1600-2800 |
| 11 | Gorebella | CIP-382173.12 | 2002 | ShARC | 1700-2400 |
| 12 | Guassa | CIP-384321.9 | 2002 | ADARC | 2000-2800 |
| 13 | Zengena | CIP-380479.6 | 2001 | AwARC | 2000-2800 |
| 14 | Zemen | AL-105 | 2001 | HU | 1700-2000 |
| 15 | Bedassa | AL-114 | 2001 | HU | 2400-3350 |
| 16 | Chiro | AL-111 | 1998 | HU | 2700-3200 |
| 17 | Wechecha | KROEZE 72-2951 | 1997 | HARC | 1700-2800 |
| 18 | Menagesha | CIP-374080.5 | 1993 | HARC | Above 2400 |
| 19 | Awash | CIP-378501.3 | 1991 | HARC | 1500-2000 |
| 20 | Alemaya 624 | AL-624 | 1987 | HU | 1700-2400 |
| 21 | Nech Abeba |  | ... | ... | Central highlands |

* ADARC= Adet Agricultural Research Centre, HU=Haramaya University, HARC=Holetta Agricultural Research Centre, AwARC= Awassa Agricultural Research Centre, ShARC= Sheno Agricultural Research Centre.
